# A Large Phyllodes Tumor With Axillary Lymph Node Involvement: A Case Report

**DOI:** 10.7759/cureus.40252

**Published:** 2023-06-11

**Authors:** Kirstie Mundok, Danielle Saldana

**Affiliations:** 1 College of Osteopathic Medicine, Edward Via College of Osteopathic Medicine, Greenville, USA; 2 General Surgery, Bon Secours Healthcare System, Greenville, USA

**Keywords:** management of phyllodes tumor, giant phyllodes tumour, malignant phyllodes, primary breast malignancy, phyllodes tumors

## Abstract

Malignant phyllodes tumors of the breast are uncommon and complex to treat. This case involves a 39-year-old woman with a rapidly growing mass in her right breast measuring 32cm. The patient underwent a bilateral mastectomy with a right sentinel node biopsy and right chest wall reconstruction. The final pathology of the tumor revealed a malignant phyllodes tumor, with one of two right axillary lymph nodes positive for metastatic phyllodes tumor. Malignant phyllodes tumors should be taken into consideration in any rapidly growing breast mass. Further studies analyzing the treatment of malignant phyllodes tumors are necessary to reduce the risk of tumor recurrence and metastasis.

## Introduction

Phyllodes tumors are rare fibroepithelial tumors that originate in the stromal tissue of the breast [1]. Phyllodes tumors are categorized into three grades: benign, borderline, and malignant [2]. The incidence of these tumors is between 0.3 and 1% of all tumors and most are benign (60-75%) [1,2]. Stromal elements are key in differentiating these tumors from fibroadenomas and determining whether they are benign or malignant [3]. These tumors are most commonly diagnosed in women between 40 and 50 years old who present with a firm, rapidly growing mass [4,5]. The median size of phyllodes tumors is 4cm, with 20% of these tumors growing larger than 10cm [6].

On ultrasound, cystic structures within a well-circumscribed solid lesion may suggest a phyllodes tumor, however, this is not sufficient to distinguish between benign or malignant phyllodes tumors and cytology is needed [7]. Another concerning obstacle with diagnosing phyllodes tumors is that only 50% of cases are correctly diagnosed on core needle biopsy [8]. Therefore, the primary treatment of phyllodes tumors is surgical excision. Local excision of benign phyllodes is curative; for intermediate tumors, excision with at least 1cm margins is utilized and these patients are still at risk for local recurrence within the first two years [8]. However, there is not a clear consensus on what constitutes a sufficient margin for malignant phyllodes [3,7,9]. Currently, they are treated similarly to sarcomas with en bloc resection; consisting of mastectomy. The requirement for lymph nodes is not typically required for this particular disease process. In malignant phyllodes tumors, adjuvant therapy remains controversial and should be administered on a case-by-case basis [3,5]. It has been postulated that there is a limited role for endocrine therapy in phyllodes tumors because the hormone receptors are primarily on the epithelial component of the tumor and not the stromal component, which is implicated in metastatic spread [9].

Local recurrence occurs at a rate of 21% and typically occurs within 2-3 years [1]. The prognosis of phyllodes tumor is an 87% 10-year survival [1]. Malignant phyllodes tumors have a risk of metastasizing hematogenously to the lungs, bones, or brain and have a poor prognosis [1].

## Case presentation

**Figure 1:**
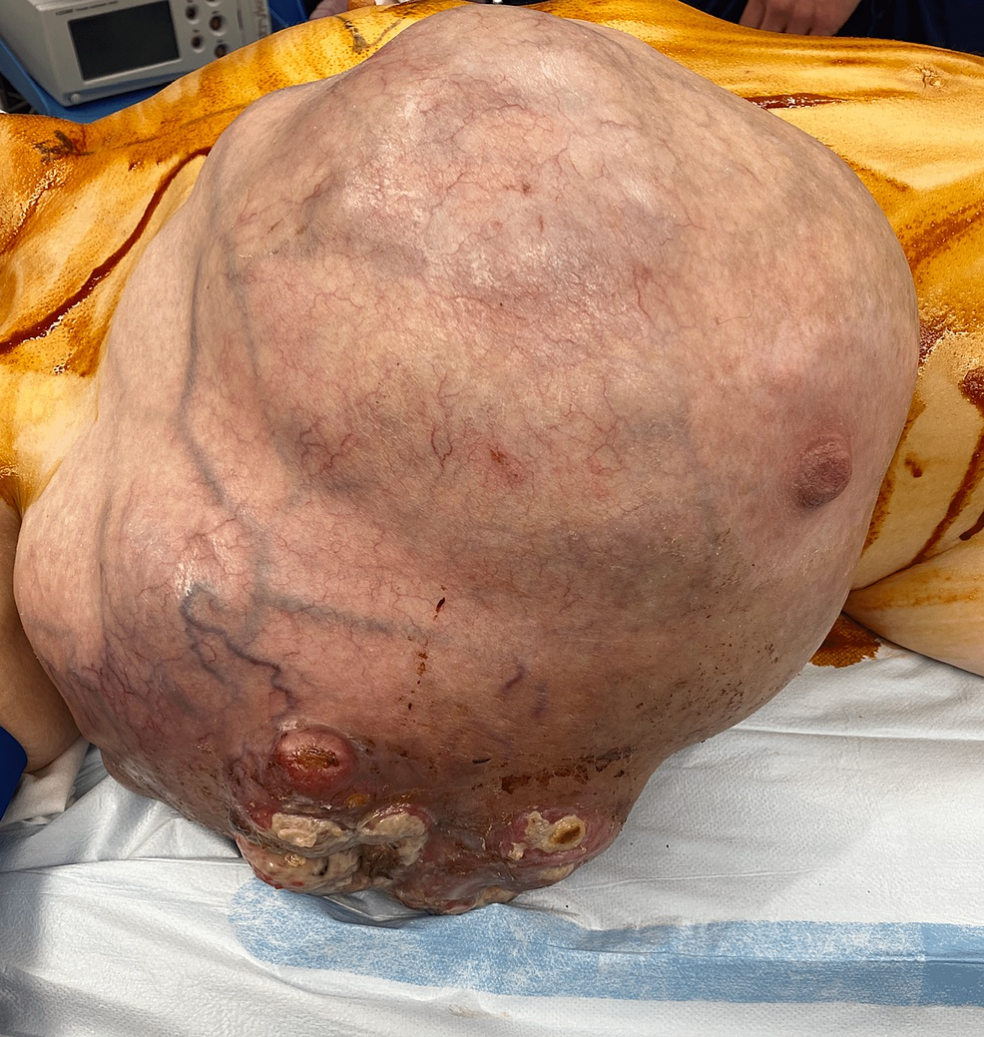
Large breast mass with skin changes on the lateral aspect.

**Figure 2:**
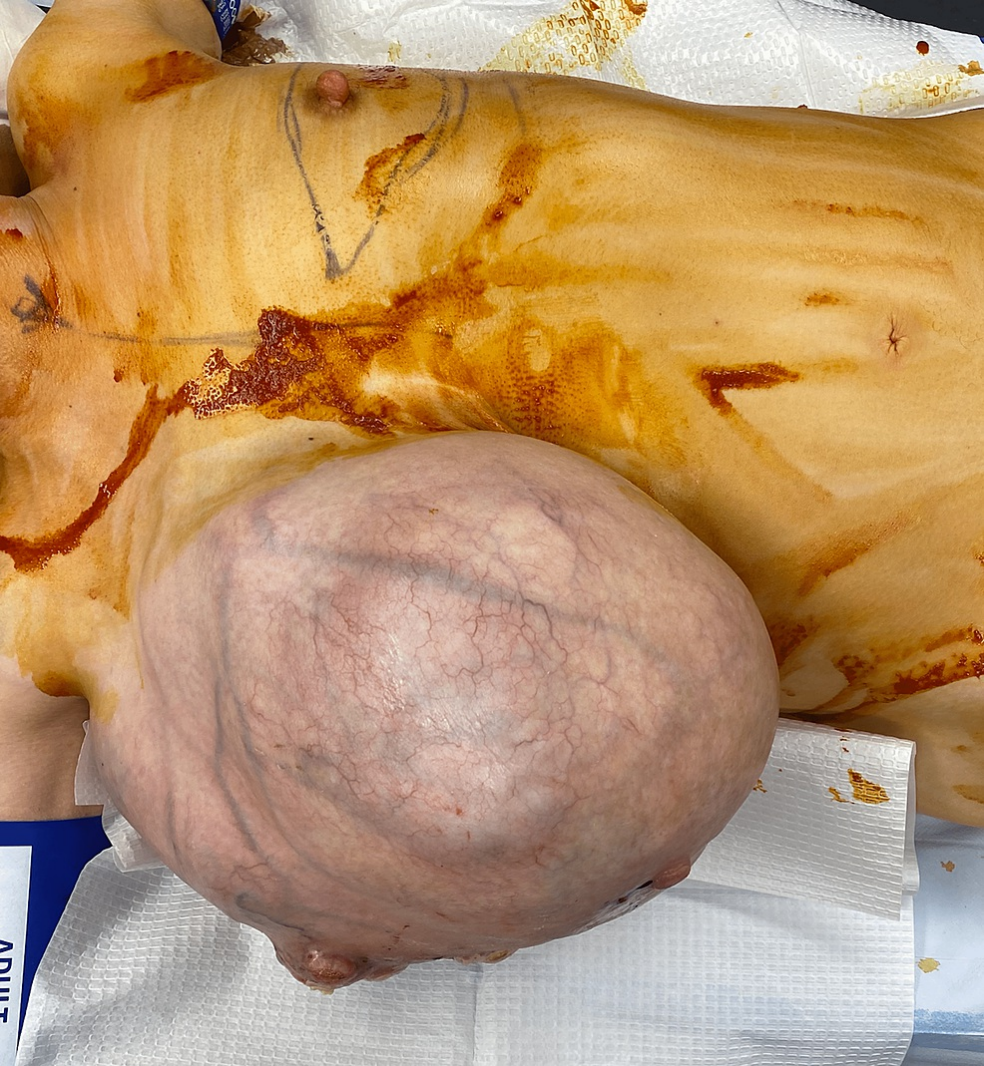
Large breast mass with skin changes on the lateral aspect.

This patient was a 39-year-old white female with a past medical history of attention deficit hyperactivity disorder, anxiety, and hypertension who presented to the general surgery clinic with a large right breast mass that she noticed three months prior to her initial visit. The patient reported that it began after suffering a chest wall injury during a race car accident, however, the area continued to enlarge. She reported that the lateral aspect of the mass began draining serous yellow fluid and blood over the last few weeks (Figures 1, 2). She did note a family history of breast cancer in her maternal and paternal grandmothers.

**Figure 3:**
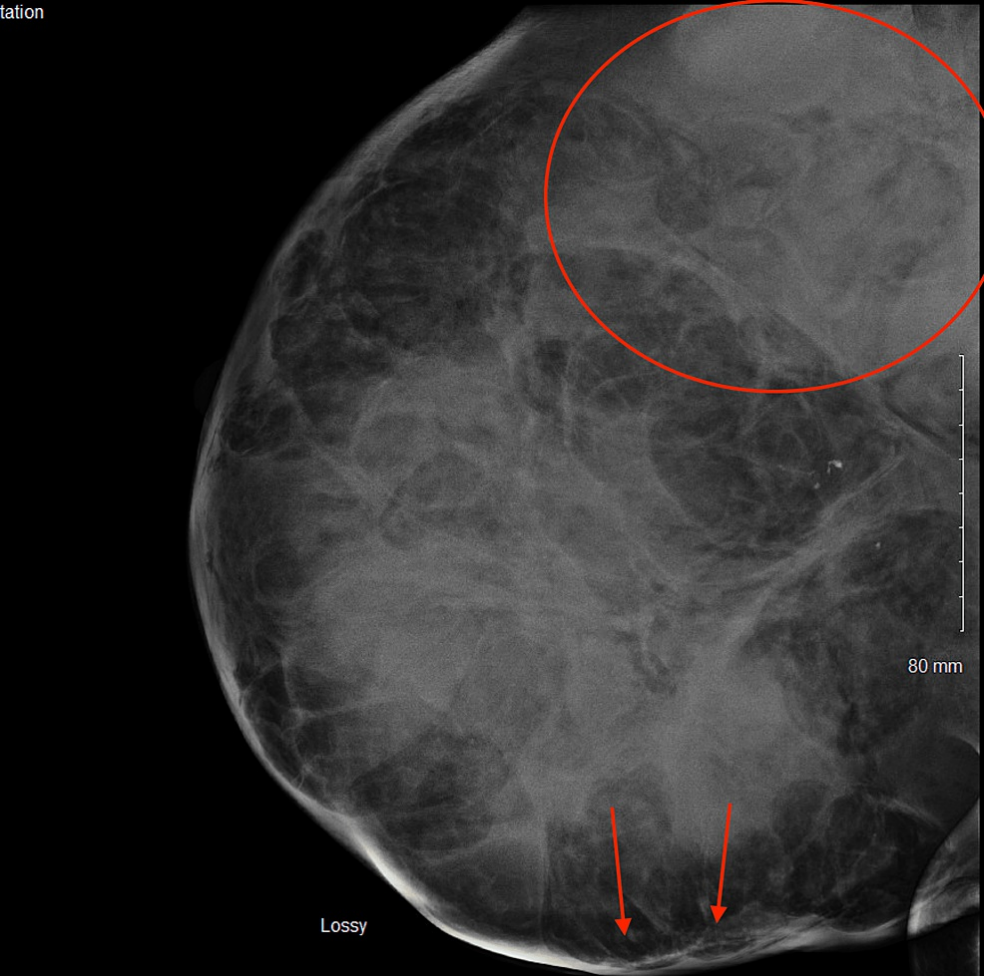
Diagnostic mammogram showing an abnormal density throughout the right breast, focally pronounced in the upper outer posterior right breast (encircled); a large superficial asymmetry was seen with changes extending to the skin surface (arrows).

The patient had an urgent bilateral diagnostic mammogram showing an abnormal density throughout the right breast, focally pronounced in the upper outer posterior right breast; a large superficial asymmetry was seen with changes extending to the skin surface (Figure 3). Ultrasound of the right breast showed a large masslike structure measuring at least 5.4cm x 6.8cm x 5.8cm in size and potentially larger. A complete blood count showed a hemoglobin of 6.7. The patient then underwent an ultrasound-guided core biopsy and outpatient packed red blood cell transfusion. Pathology revealed a malignant spindle cell neoplasm. Differential diagnoses included a malignant phyllodes tumor and inflammatory breast cancer.

**Figure 4:**
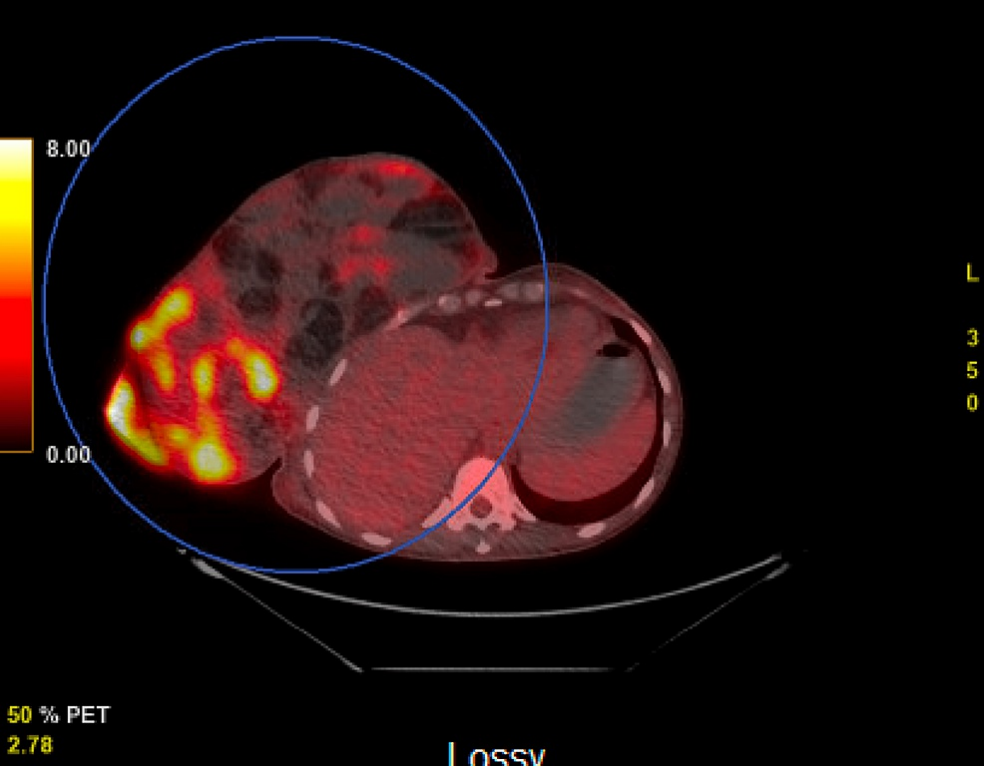
Positron emission tomography (PET) scan revealing a large heterogeneous soft tissue and fatty mass extending from the right breast demonstrating areas of hypermetabolic activity.

**Figure 5:**
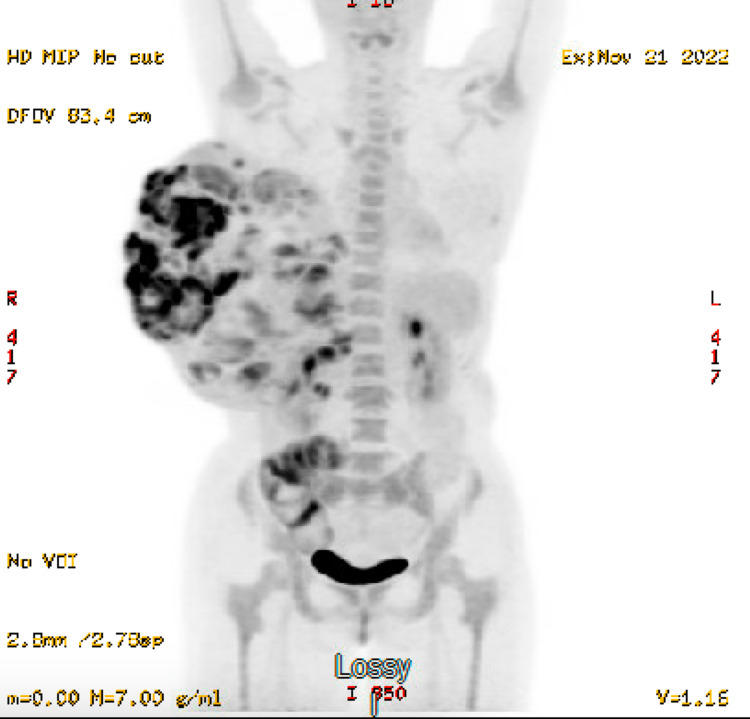
Positron emission tomography (PET) scan revealing a large heterogeneous soft tissue and fatty mass extending from the right breast demonstrating areas of hypermetabolic activity with a centrally cystic or necrotic mediastinal lesion noted. There was no evidence of metastatic disease within the abdomen and pelvis.

A positron emission tomography (PET) scan was then obtained revealing a large heterogeneous soft tissue and fatty mass extending from the right breast demonstrating areas of hypermetabolic activity with a centrally cystic or necrotic mediastinal lesion noted (Figures 4, 5). There was no evidence of metastatic disease within the abdomen and pelvis.

The patient agreed to proceed with a bilateral mastectomy with right sentinel node biopsy and simultaneous right chest wall reconstruction. The patient underwent a muscle flap closure with a split-thickness skin graft from her right lower extremity to the chest. We elected to do a sentinel lymph node dissection due to the overall size of the mass. Unfortunately, due to the size of the mass the Technecium-99 was not picking up preoperatively. During the procedure, we elected to take any palpable lymph nodes for sampling. Final pathology from the right breast showed a malignant phyllodes tumor, measuring 32cm in greatest dimension with margins negative for tumor and benign skin and nipple. One of two right axillary lymph nodes was positive for metastatic phyllodes tumor. The patient received five units of packed red blood cells prior to discharge from the hospital due to a drop in hemoglobin to 5.9. The patient recovered well postoperatively.

This patient is currently following up with oncology to develop a treatment plan as this is a rare tumor involving an axillary lymph node, along with questionable further disease in the mediastinum as shown on the PET scan. It is unclear if this patient will benefit from radiation at this time as she will need further assessment of her overall disease status.

## Discussion

Phyllodes tumors are rare breast tumors that can have various presentations. Phyllodes tumors make up less than 1% of all breast neoplasms and are typically benign [10]. There are no definitive features indicating a phyllodes tumor on mammogram or ultrasound, which makes the differentiation between a phyllodes tumor and fibroadenoma challenging [10,11].

Axillary lymph node involvement is rare in phyllodes tumors. In fact, routine axillary dissection is not recommended in phyllodes tumors [10]. Few cases of phyllodes tumors involving an axillary lymph node have been reported in the literature. In a series of previous studies, the incidence of axillary lymph node involvement in a cystosarcoma phyllodes tumor was between 1 and 4% [12-14]. The staging of phyllodes tumors typically follows that of a sarcoma in the breast with the mainstay of therapy being surgical excision and radiation [15].

## Conclusions

This was an unusual case of a very large phyllodes tumor with axillary lymph node involvement. Although uncommon, phyllodes tumors do have the potential to spread to lymph nodes and other organs. This should be taken into consideration when a breast abnormality is found on a self-exam, ultrasound, or mammogram. Additional research may look into the differences between fibroadenomas and phyllodes tumors in imaging studies to prevent the rapid expansion of these tumors.

## References

[1] Limaiem F, Kashyap S (2022). Phyllodes Tumor of the Breast. https://www.ncbi.nlm.nih.gov/books/NBK541138/.

[2] Tse GM, Niu Y, Shi HJ (2010). Phyllodes tumor of the breast: an update. Breast Cancer.

[3] Demian GA, Fayaz S, El-Sayed Eissa H (2016). Phyllodes tumors of the breast: analysis of 35 cases from a single institution. J Egypt Natl Canc Inst.

[4] Ditsatham C, Chongruksut W (2019). Phyllodes tumor of the breast: diagnosis, management and outcome during a 10-year experience. Cancer Manag Res.

[5] Simpson A, Li P, Dietz J (2021). Diagnosis and management of phyllodes tumors of the breast. Ann Breast Surg.

[6] Liang MI, Ramaswamy B, Patterson CC, McKelvey MT, Gordillo G, Nuovo GJ, Carson WE 3rd (2008). Giant breast tumors: surgical management of phyllodes tumors, potential for reconstructive surgery and a review of literature. World J Surg Oncol.

[7] Wei Y, Yu Y, Ji Y (2022). Surgical management in phyllodes tumors of the breast: a systematic review and meta-analysis. Gland Surg.

[8] Townsend JCM, Beauchamp RD, Evers BM, Mattox KL (2016). Sabiston Textbook of Surgery.

[9] Tan BY, Acs G, Apple SK (2016). Phyllodes tumours of the breast: a consensus review. Histopathology.

[10] Shafi AA, AlHarthi B, Riaz MM, AlBagir A (2020). Giant phyllodes tumor with axillary & interpectoral lymph node metastasis; a rare presentation. Int J Surg Case Rep.

[11] Buchberger W, Strasser K, Heim K, Müller E, Schröcksnadel H (1991). Phylloides tumor: findings on mammography, sonography, and aspiration cytology in 10 cases. AJR Am J Roentgenol.

[12] Treves N (1964). A study of cystosarcoma phyllodes. Ann N Y Acad Sci.

[13] Norris HJ, Taylor HB (1967). Relationship of histologic features to behavior of cystosarcoma phyllodes - analysis of ninety-four cases. Cancer.

[14] Staren ED, Lynch G, Boyle C, Witt TR, Bines SD (1994). Malignant cystosarcoma phyllodes. Am Surg.

[15] Li GZ, Raut CP, Hunt KK, Feng M, Chugh R (2021). Breast sarcomas, phyllodes tumors, and desmoid tumors: epidemiology, diagnosis, staging, and histology-specific management considerations. Am Soc Clin Oncol Educ Book.

